# Outcomes of linezolid, glycopeptides, and other regimens in ampicillin-resistant, vancomycin-susceptible and vancomycin-resistant *enterococcal* bacteremia in a resource-limited setting: stewardship implications

**DOI:** 10.1007/s00210-026-05701-2

**Published:** 2026-07-12

**Authors:** Yameena Noman Khan, Ali Mala, Rubab Murtaza, Irshad Batool, Azizullah Khan, Imtiaz Begum, Jamil Muqtadir, Abdul Rasheed, Sameeullah Bhatti

**Affiliations:** 1 Department of Internal Medicine, Dr. Ziauddin Hospital, Allama Rasheed Turbani Rd, Waheed Colony, Karachi, 75000 Sindh Pakistan; 2 Department of Gastroenterology, Aga Khan University Hospital, Stadium Road, Karachi, Sindh Pakistan; 3 Department of Infectious Disease, Dow University of Health Sciences, New Labor Colony Nanakwara, Mission Rd, Karachi, 74200 Sindh Pakistan; 4 Department of Cardiology, National Institute of Cardiovascular Diseases, Rafiqui (H.J.), Shaheed Road, Karachi, 75510 Sindh Pakistan; 5 Department of Critical Care, Mount Sinai Hospital, New York, NY USA; 6 Department of Infectious Disease, University at Buffalo, Buffalo, USA

**Keywords:** Vancomycin-resistant *Enterococcus*, Vancomycin sensitive *Enterococcus*, Linezolid, Mortality, ICU admission

## Abstract

*Enterococcal* bloodstream infections (BSI) are associated with high morbidity and limited treatment options, particularly in resource-limited settings where access to standard agents may be constrained. Comparative effectiveness data directly informing the choice among available agents for both vancomycin-resistant (VRE) and ampicillin-resistant, vancomycin-susceptible (VSE) *enterococcal* bacteremia are specifically lacking in such settings, where regimen selection is frequently dictated by drug availability and cost rather than guideline preference. We conducted a retrospective cohort study across three hospitals of a single university health system (Dr. Ziauddin University, Karachi, Pakistan) including hospitalized adults (≥ 18 years) with clinically significant *enterococcal* bacteremia (a positive blood culture together with clinical evidence of infection, as adjudicated by the treating physician) between January 1, 2010, and May 31, 2025. Only the first episode per patient was analyzed. VRE and VSE cohorts were compared for baseline characteristics and empiric therapy. Definitive monotherapy—receipt of a single study agent active against the isolate, assigned within the prespecified 48- to 72-h window after index blood culture (time-zero), by which time organism identification and susceptibility results were generally available—was assessed within the VRE and VSE cohorts. Because discharge alive is a competing event for in-hospital death, cumulative incidence functions (CIF) with Gray’s test and an adjusted Fine–Gray competing-risks model (adjusted for age, Charlson Comorbidity Index, qPitt score, septic shock, and definitive antibiotic exposure (vancomycin, teicoplanin, daptomycin, tigecycline)) were used. Of 925 screened patients, 501 met inclusion criteria (VSE *n* = 404; VRE *n* = 97). VRE bacteremia presented with greater acute severity (higher APACHE II) and was more frequently catheter-associated, while VSE more often had urinary and intra-abdominal sources (all *p* < 0.001). In definitive monotherapy analyses among VRE (*n* = 67; tigecycline excluded, as it was used only as salvage therapy), 28-day mortality was numerically higher with teicoplanin (66.7%) but did not differ significantly across linezolid, teicoplanin, and daptomycin; CIF analysis similarly showed no difference in cumulative incidence of in-hospital death (Gray’s *p* = 0.246). Tigecycline, used only as salvage therapy, was associated with higher 28-day mortality (80.0% vs 47.8%; *p* = 0.003; Supplementary Table S1). In VSE monotherapy (*n* = 404), teicoplanin exposure was associated with higher ICU admission and higher 28-day mortality, and CIF curves differed across vancomycin, teicoplanin, and linezolid (Gray’s *p* < 0.001); however, the linezolid-exposed group had sparse/zero events for several endpoints, suggesting confounding by indication. In the adjusted Fine–Gray model, higher qPitt score was independently associated with mortality (sHR 2.21, 95% CI 1.62–3.01; *p* < 0.001). Tigecycline (sHR 4.00, 95% CI 2.11–7.60; *p* < 0.001) and daptomycin exposure (sHR 3.11, 95% CI 1.37–7.07; *p* = 0.007) were associated with higher subdistribution hazard of death, whereas vancomycin exposure was associated with lower hazard (sHR 0.36, 95% CI 0.20–0.67; *p* = 0.001). In this multi-hospital, single-system cohort, VRE bacteremia was more severe and more often catheter-associated than VSE. When accounting for discharge as a competing event, mortality differences across definitive monotherapy groups were not significant in VRE, while VSE comparisons were influenced by treatment selection and sparse outcome counts in some groups. Severity (qPitt score) was a key independent predictor of mortality, and observed antibiotic–mortality associations should be interpreted cautiously given confounding by indication.

**Supplementary information:**

The online version contains supplementary material available at https://doi.org/10.1007/s00210-026-05701-2.

## Introduction

*Enterococci*—specifically *Enterococcus faecium* and *Enterococcus faecalis*—are among the most commonly identified health care-associated pathogens, although *E. faecalis* is significantly more prevalent (80–90% of clinical *enterococcal* isolates), with *E. faecium* a distant second (Murray 1990). Gut colonization with *enterococci* is common in critically ill patients exposed to prolonged antibiotic therapy and can serve as a reservoir for subsequent bloodstream infection (Arias 2012). Many species of *enterococci—*especially *E. faecalis*—have innate resistance mechanisms and are therefore intrinsically resistant to many antibiotics. For this reason, ampicillin or penicillin are considered first-line treatments when the *enterococcal* isolate is sensitive to them; vancomycin is recommended when there is evidence of high-level beta-lactam resistance or when there is a contraindication (Choice of antibacterial drugs 2007). Because vancomycin-resistant *enterococci* (VRE) have been shown to exhibit low susceptibility to many other antibiotics in cases where they develop resistance to vancomycin and beta-lactams, the primary drugs used to treat VRE infections include linezolid and daptomycin (Barber 2015; Wang 2009). It has also been demonstrated that daptomycin exhibits bactericidal activity in vitro against VRE (Heidary 2018). A number of studies have evaluated and compared these two agents as a potential treatment for VRE bacteremia (Twilla et al. 2012; Chou et al. 2012; Chuang et al. 2016; Ye et al. 2018; Britt et al. 2015); however, the best therapeutic approach remains unknown. Linezolid, a bacteriostatic oxazolidinone, has been approved by the US Food and Drug Administration (FDA) for use to treat VRE infections Hashemian 2018. Although daptomycin is used as an “off-label” treatment for VRE infections, it has not been FDA-approved to treat VRE bacteremia (Heidary 2018). Although not FDA-approved for any bloodstream infection, tigecycline retains in vitro activity against the majority of VRE isolates, with reported MICs against *E. faecium* lower than those for *E. faecalis* and *staphylococci* (Waites et al. 2006; Lefort et al. 2003; Murphy et al. 2000). Systematic reviews with meta-analyses have indicated that linezolid treatment for VRE bacteremia was associated with a significant reduction in mortality compared to daptomycin treatment; however, all of the studies included in these analyses were retrospective and highly heterogeneous (Whang et al. 2013; Balli et al. 2014; Chuang 2014). For bacteremia caused by ampicillin-resistant, vancomycin-susceptible *Enterococcus* (VSE), appropriate therapy options include glycopeptide inhibitors or daptomycin. However, there is no data on the use of linezolid for the treatment of VSE BSI. Similarly, standard of care for VRE remains linezolid and daptomycin, but there is limited data on teicoplanin and tigecycline. In a country like Pakistan, there are limited treatment options for VRE. Linezolid remains the standard of care, since daptomycin is not available in most of the instances; therefore, clinicians decide the alternative drugs based on the availability even if not standard of care.

This study examined adult patients at our hospital with *enterococcal* BSI. Accordingly, we sought to characterize the clinical features and outcomes of *enterococcal* bloodstream infections at our center, identify independent predictors of mortality in VRE and VSE bacteremia, and evaluate the association between antibiotic choice—particularly linezolid use and nonstandard regimens used in resource-limited settings—and patient outcomes.

## Methods

### Study designs, setting, and population

The retrospective study was performed over 15 years from January 1, 2010, to May 31, 2025, at Dr. Ziauddin University’s three hospitals in Karachi, Pakistan. All patients aged 18 years or greater who had at least one *Enterococcus*-positive blood culture during their hospitalization were eligible for inclusion. Only patients who met the criteria for clinically significant *enterococcal* bacteremia (positive blood cultures and clinical evidence of an infectious process, as adjudicated by the treating physician) were enrolled in this study. For patients with recurrent episodes of *enterococcal* bacteremia, only the first episode was included in the study (Noskin 1995). We sought to eliminate the bias that is present in decision-making related to antibiotics because of changes over time in the selection of antibiotic therapy. To do this, we created a comparison of initial treatment strategies using a prespecified early treatment window (first 48–72 h after time-zero) and defined treatment categories based on the agent(s) administered during that window, using mutually exclusive group definitions as specified below. In this regard, the time of collection of the first blood culture (“index”) was designated as “time-zero” for all analyses evaluating time to an event (i.e., time-to-event analyses). By using this method, we could anchor all follow-up at “time-zero” and assign the timing of when a specific antibiotic was administered within an early window to eliminate or minimize the potential for immortal time bias (Suissa 2008), which can occur if the individual has to survive long enough before being able to be treated with a specific antibiotic.

### Exclusion criteria

Individuals under 18 years of age, people whose medical records are incomplete, and anyone with a non-*enterococcal* bloodstream infection or with polymicrobial bacteremia defined as a second pathogen isolated from blood within 3 calendar days before or after the index *enterococcal* blood culture, were excluded from the study. Patients were excluded if none of the study antibiotics (linezolid, tigecycline, teicoplanin, daptomycin, or vancomycin) were initiated within the first 48–72 h after index blood culture collection (time-zero). We also excluded the patients who isolated ampicillin-sensitive *Enterococcus.*

### Identification and susceptibility testing

The BD/BACTEC/9000 system was used to perform blood cultures. Traditional biochemical methods were utilized to ascertain the isolates’ identities. According to the Clinical and Laboratory Standards Institute (CLSI) recommendations, Mueller Hinton Agar (MHA) medium was used for the modified Kirby–Bauer disk diffusion method for conducting a susceptibility test for *E. *species (Gray 1994). Minimum inhibitory concentrations (MICs) were interpreted according to CLSI criteria: vancomycin susceptibility was defined as MIC ≤ 4 µg/mL (resistant ≥ 32 µg/mL), linezolid as susceptible ≤ 2 µg/mL (intermediate 4 µg/mL; resistant ≥ 8 µg/mL), and daptomycin as susceptible-dose dependent with MIC ≤ 4 µg/mL for *Enterococcus* spp. EUCAST clinical breakpoints were used for teicoplanin susceptible ≤ 2 mg/L, and tigecycline susceptible ≤ 0.5 mg/L (Gupta 2015). Vitek-2 method was employed for MIC, except daptomycin which required *E*-test. MIC data was available for a number of isolates across the study time frame, specifically, MIC data was available for linezolid, tigecycline and daptomycin (*E*-test) in the VRE cohort and for linezolid, vancomycin and teicoplanin in the VSE cohort. Where MIC data were unavailable, the analysis was based on categorical susceptibility results and MIC missingness is reported. Among VRE isolates, teicoplanin susceptibility by disk diffusion was used as a phenotypic surrogate for VanB-type resistance; VanA/VanB genotyping and species-level identification were not available. We additionally abstracted exact timing of blood culture collection and antibiotic initiation, empiric regimens started within the first 48–72 h, and subsequent regimen switching (agent-to-agent transitions and timing). We also extracted adverse events relevant to regimen selection, including thrombocytopenia and acute kidney injury.

### Data collection

All patients admitted with *enterococcal* BSI had their electronic medical records gathered. Age, gender, comorbidities such as diabetes mellitus, ischemic heart disease, hematological malignancies or solid organ tumors, smoking, laboratory results, and clinical outcomes were all thoroughly reviewed in the patient’s chart. A manual review of the patient’s charts was also carried out as part of the process to guarantee accurate data collection.

### Clinical conditions, treatment, and outcome

Information was gathered about the patient’s vital signs, mechanical ventilation, length of stay in the intensive care unit (ICU), neutropenia (absolute neutrophil count less than 500 cells/mm^3^) (Putnam 2010), source and time to blood-culture positivity of *enterococcal* growth. All patients treated with linezolid 600 mg twice daily, teicoplanin 12 mg/kg q 12 h on days 1 and 2, then 12 mg/kg q 24 h, tigecycline 200 mg bolus, followed by 100 mg q 12 h, daptomycin 10 mg/kg q24h, and vancomycin 25–30 mg/kg/day loading dose, followed by 15–20 mg/kg/day initiated within the first 48–72 h after index blood culture collection (time-zero). Choice of antibiotics followed institutional practices, drug availability, and cost. For VRE, the preferred drug remained linezolid as daptomycin is rarely available for use. For patients with teicoplanin sensitive VRE, institutional recommendations prefer the use of teicoplanin in cases of the unavailability of daptomycin. But, tigecycline was used as salvage therapy in cases with contraindications precluding the use of linezolid and had no other available treatment options. For VSE, the preferred drug remained vancomycin with teicoplanin and linezolid used alternatively.

The study defines exposure groups based on initial treatment strategies. The study defines these exposure groups as non-overlapping and mutually exclusive treatment options based on the first 48–72 h post time zero for both VRE and VSE. For VRE bacteremia, the initial treatment strategy options were linezolid, high-dose daptomycin (≥ 9 mg/kg/day), teicoplanin (for strains that were susceptible to teicoplanin and thus serve as a phenotypic indicator for VanB type activity (Riccardi 2021), and tigecycline (treated as salvage and thus analyzed separately). For VSE bacteremia, the initial treatment strategy options were vancomycin, teicoplanin, and linezolid. The frequency, timing, and direction of the regimen changes during the first 48- to 72-h period were documented and used in sensitivity analysis. Definitive monotherapy was defined as treatment with exactly one study agent to which the isolate was susceptible, assigned within the prespecified 48- to 72-h window after index blood culture (time-zero), by which time organism identification and susceptibility results were generally available. Patients receiving more than one study agent within this window were not included in the definitive monotherapy analyses.

The primary outcome was in-hospital mortality within 28 days, defined as death occurring during the index hospitalization within 28 days of index blood culture collection (time-zero). Time-zero for all time-to-event analyses was the blood culture collection time, and exposure was assigned within the prespecified 48–72-h window to reduce immortal time bias. The secondary outcomes were clinical failure and microbiological failure at 7 days. Additional secondary outcomes were length of hospital stay; requirement for an Intensive care unit (ICU) admission and/or requirement for Invasive Mechanical Ventilation (IMV). We also evaluated in-hospital mortality as an outcome in which discharge alive was treated as a competing event.

### Operational definitions

Microbiological failure: defined as persistent positive blood cultures on or after day 7 from the index blood culture.

Clinical failure was defined as persistent bacteremia on or after day 7 from time-zero and/or persistent fever > 38.3 °C on or after day 7. ICU admission and invasive mechanical ventilation were analyzed as separate outcomes rather than components of the clinical failure definition.

Thrombocytopenia was defined as a platelet count < 100 × 10⁹/L or a ≥ 20% decline from baseline after antibiotic initiation (whichever occurred first) during hospitalization (Johansen et al. 2013). Acute kidney injury (AKI) was defined as an increase in serum creatinine ≥ 0.3 mg/dL within 48 h or ≥ 50% from baseline during hospitalization (Pereira et al. 2017).

### Statistical analysis

We conducted all statistical testing using IBM SPSS Statistics version 27. For continuous variables with approximately normal distributions, we presented the mean (standard deviation); for those without approximately normal distributions, we used the median (interquartile range). To compare the groups (VRE vs VSE), we used an independent-samples *t*-test to compare the means of normally distributed continuous variables and a Mann–Whitney *U* test for non-normally distributed continuous variables. For categorical variables, we reported counts (with percentages) and compared them using the Chi-Square test or Fisher’s Exact test, as appropriate.

For within-cohort comparisons of definitive monotherapy outcomes, we reported counts (%) and unadjusted comparisons using Chi-square or Fisher’s exact test as appropriate. Because discharge alive is a competing event for in-hospital death, we estimated cumulative incidence functions (CIFs) and compared groups using Gray’s test. We then fit an adjusted Fine–Gray subdistribution hazards model for in-hospital mortality, treating discharge alive as a competing event (fstatus: 1 = death, 2 = discharge, cencode = 0). CIF/Gray and Fine–Gray analyses were restricted to patients receiving exactly one definitive agent; tigecycline was excluded from VRE regimen comparisons. Missing data was handled using complete case analysis (Sterne et al. 2009; Little et al. 2012).

## Results

**Fig. 1:**
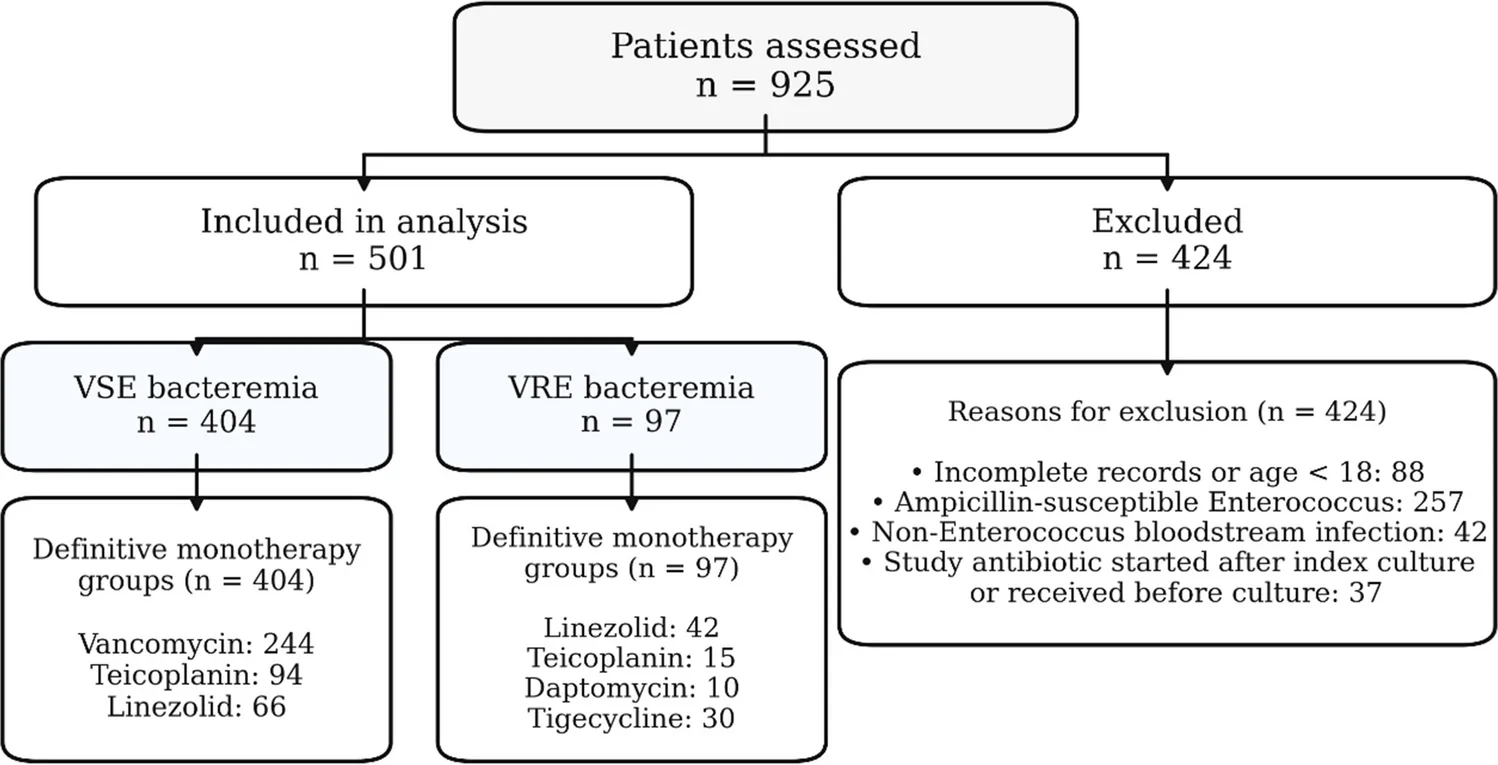
Flow diagram of patients included in the study

Of 925, a total of 501 patients were included in this study (Fig. 1). Demographics in accordance with the type of bacteremia are shown in Table 1. In baseline comparisons, VRE bacteremia presented with greater acute illness severity than VSE, reflected by a higher APACHE II score (14.59 ± 5.32 vs 12.49 ± 1.82; *p* < 0.001), while age and most comorbidities were similar between groups, including Charlson Comorbidity Index (*p* = 0.310). Two comorbidity differences stood out: hematologic malignancy was more frequent in VRE (12.4% vs 5.2%; *p* = 0.011), whereas asthma/COPD was less common in VRE (1.0% vs 8.7%; *p* = 0.009). The source of bacteremia differed markedly (*p* < 0.001): VRE was disproportionately associated with an intravascular catheter source (38.1% vs 16.1%), whereas VSE more often had urinary tract (34.7% vs 25.8%) and intra-abdominal/GI sources (10.6% vs 3.1%). Laboratory differences were statistically significant but modest in magnitude: VRE had slightly higher median hemoglobin (11.20 vs 10.70 g/dL; *p* = 0.028) and higher median WBC count (13.20 vs 12.0; *p* = 0.003), along with small elevations in bilirubin (*p* = 0.031) and ALT (*p* = 0.004). Other parameters (platelets, INR/PTT, creatinine, sodium, bicarbonate) were not meaningfully different.

Initial empiric antibiotic regimens initiated within 48–72 h of index blood culture collection are summarized in Table 2. Piperacillin–tazobactam was used more frequently in VSE than VRE bacteremia (35.6% vs 13.4%; *p* < 0.001), whereas carbapenem therapy was more common in VRE than VSE bacteremia (41.2% vs 19.8%; *p* < 0.001). Use of ceftriaxone and amoxicillin–clavulanic acid did not differ significantly between cohorts, and combination regimens with vancomycin showed no statistically significant differences between groups.

**Table 1:** Baseline characteristics based on VRE/VSE bacteremia

Variable	Total, *N* = 501 (%)	VSE, bacteremia *N* = 404 (%)		VRE bacteremia *N* = 97 (%)	*p*-value
Age (mean ± SD)	53.66 ± 18.44	54.12 ± 18.47		51.78 ± 18.30	0.263
APACHE II score at presentation (Mean ± SD)	12.90 ± 2.97	12.49 ± 1.82		14.59 ± 5.32	< 0.001
Male	289 (57.7)	232 (57.4)		57 (58.8)	0.811
Diabetes	227 (45.3)	186 (46.0)		41 (42.3)	0.503
Hypertension	326 (65.1)	270 (66.8)		56 (57.7)	0.091
Asthma–chronic obstructive pulmonary disease	36 (7.2)	35 (8.7)		1 (1.0)	0.009
Solid organ malignancy	47 (9.4)	40 (9.9)		7 (7.2)	0.415
Hematologic malignancy	33 (6.6)	21 (5.2)		12 (12.4)	0.011
Chronic liver disease	43 (8.6)	37 (9.2)		6 (6.2)	0.348
Ischemic heart disease	189 (37.7)	156 (38.6)		33 (34)	0.402
Charlson comorbidity index	3.24 ± 2.85	3.31 ± 2.91		2.98 ± 2.56	0.310
Smoking	72 (14.4)	56 (13.9)		16 (14.4)	0.507
Primary source of infection					< 0.001
Urinary tract infection	165 (32.9)	140 (34.7)		25 (25.8)	
Intra-abdominal/gastrointestinal	46 (9.2)	43 (10.6)		3 (3.1)	
Cutaneous wounds	23 (4.6)	23 (5.7)		0 (0)	
Intravascular catheters	102 (20.4)	65 (16.1)		37 (38.1)	
Respiratory	64 (12.8)	49 (12.1)		15 (15.5)	
Pelvic	26 (5.2)	19 (4.7)		7 (7.2)	
Unknown	75 (15.0)	65 (16.1)		10 (10.3)	
Laboratory parameters, median (IQR)					
Hemoglobin (gm/dL)	10.90 (9.50–12.60)	10.70 (9.50–12.50)		11.20 (9.65–13.0)	0.028
White blood cells (/µL)	12.80 (9.0–17.90)	12.0 (8.70–16.07)		13.20 (10.50–20.25)	0.003
Platelets (/µL)	193 (115–285)	189 (118–279)		212 (110–305)	0.928
Partial thromboplastin time (sec)	35 (30.40–40.0)	35 (30.2–40.6)		35 (31.05–39)	0.825
International normalized ratio	1.10 (1.0–1.30)	1.10 (1.0–1.30)		1.06 (1.0–1.43)	0.768
Total bilirubin (mg/dL)	0.90 (0.57–1.50)	0.83 (0.54–1.40)		1.10 (0.60–1.70)	0.031
Serum alanine aminotransferase (ALT) (/L)	36 (29–67)	35 (29–70.5)		45 (32–67)	0.004
Creatinine (mg/dL)	1.41 (0.95–3.40)	1.40 (0.90–3.10)		1.44 (0.98–3.14)	0.598
Sodium (M Eq/L)	136 (132–139)	136 (132–140)		136 (131–139)	0.075
Bicarbonate (M Eq/L)	22 (18–25)	22 (18–25)		22.30 (17.1–26.95)	0.617

*SD* standard deviation, *VRE* vancomycin-resistant *Enterococcus*, *VSE* vancomycin-sensitive *Enterococcus*

**Table 2:** Empiric antibiotics administered prior to availability of blood culture results, by bacteremia type (VSE vs VRE)

Variable	Total, *N* = 501 (%)	VSE bacteremia, *N* = 404 (%)	VRE bacteremia, *N* = 97 (%)	*p*-value
Ceftriaxone	52 (10.4)	38 (9.4)	14 (14.4)	0.145
Amoxicillin clavulanic acid	66 (13.2)	54 (13.4)	12 (12.4)	0.795
Piperacillin tazobactam	157 (31.3)	144 (35.6)	13 (13.4)	< 0.001
Carbapenem	120 (24)	80 (19.8)	40 (41.2)	< 0.001
Carbapenem + vancomycin	33 (6.6)	31 (7.7)	2 (2.1)	0.064
Piperacillin tazobactam + vancomycin	73 (14.6)	57 (14.1)	16 (16.5)	0.550

*VSE* vancomycin-sensitive *Enterococcus*, *VRE* vancomycin-resistant *Enterococcus*

Among 67 VRE patients (Table 3) who received only one definitive study drug (linezolid *n* = 42, teicoplanin *n* = 15, daptomycin *n* = 10), 28-day mortality was numerically highest with teicoplanin (66.7% vs 42.3% in those not receiving teicoplanin; *p* = 0.096), while no significant mortality differences were observed for daptomycin (50.0% vs 47.4%; *p* = 0.878) or linezolid (40.5% vs 60.0%; *p* = 0.122). Rates of AKI and thrombocytopenia did not differ significantly by drug exposure (AKI: teicoplanin *p* = 0.147, daptomycin *p* = 0.151, linezolid *p* = 0.849; thrombocytopenia: teicoplanin *p* = 0.224, daptomycin *p* = 0.129, linezolid *p* = 0.943). ICU admission and septic shock were also not significantly different across comparisons (all *p* > 0.05). Intubation showed a nonsignificant trend toward higher rates with daptomycin (70.0% vs 38.6%; *p* = 0.065), while teicoplanin (*p* = 0.764) and linezolid (*p* = 0.105) were not associated with significant differences.

**Table 3:** VRE bacteremia (tigecycline excluded): outcomes by definitive monotherapy (*n* = 67)

Outcome	Teicoplanin, no (*n* = 52)	Teicoplanin, yes (*n* = 15)	Daptomycin, no (*n* = 57)	Daptomycin, yes (*n* = 10)	Linezolid, no (*n* = 25)	Linezolid, yes (*n* = 42)
28-day mortality	22 (42.3%)	10 (66.7%)	27 (47.4%)	5 (50.0%)	15 (60.0%)	17 (40.5%)
Acute kidney injury	6 (11.5%)	4 (26.7%)	10 (17.5%)	0 (0.0%)	4 (16.0%)	6 (14.3%)
Thrombocytopenia	7 (13.5%)	4 (26.7%)	11 (19.3%)	0 (0.0%)	4 (16.0%)	7 (16.7%)
Septic shock	6 (11.5%)	4 (26.7%)	10 (17.5%)	0 (0.0%)	4 (16.0%)	6 (14.3%)
Intensive care unit admission	16 (30.8%)	3 (20.0%)	41 (71.9%)	7 (70.0%)	19 (76.0%)	29 (69.0%)
Intubation	22 (42.3%)	7 (46.7%)	22 (38.6%)	7 (70.0%)	14 (56.0%)	15 (35.7%)

In the VSE cohort (Table 4), teicoplanin exposure was associated with higher ICU admission (25.5% vs 14.8%; *p* = 0.016) and higher 28-day mortality (16.0% vs 4.2%; *p* < 0.001). Vancomycin exposure was strongly associated with AKI (25.8% vs 6.9%; *p* < 0.001), while other vancomycin comparisons were not significant (ICU *p* = 0.317; mortality *p* = 0.117). In contrast, the linezolid-exposed group had zero events for ICU admission, septic shock, AKI, intubation, and mortality, so the statistically significant differences seen for these outcomes (ICU *p* < 0.001; septic shock *p* = 0.002; mortality *p* = 0.015; AKI *p* < 0.001; intubation *p* = 0.033) likely reflect sparse/structural zero counts and strong confounding by indication (i.e., linezolid preferentially used in lower-acuity patients) rather than a definitive protective effect; thrombocytopenia did not differ (*p* = 0.700).

**Table 4:** VSE bacteremia: outcomes by definitive monotherapy (*n* = 404)

Variable	Teicoplanin, no (*n* = 310)	Teicoplanin, yes (*n* = 94)	Vancomycin, no (*n* = 160)	Vancomycin, yes (*n* = 244)	Linezolid, no (*n* = 338)	Linezolid, yes (*n* = 66)
Intensive care unit admission	46/310 (14.8%)	24/94 (25.5%)	24/160 (15.0%)	46/244 (18.9%)	70/338 (20.7%)	0/66 (0.0%)
Septic shock	31/310 (10.0%)	11/94 (11.7%)	11/160 (6.9%)	31/244 (12.7%)	42/338 (12.4%)	0/66 (0.0%)
28-day mortality	13/310 (4.2%)	15/94 (16.0%)	15/160 (9.4%)	13/244 (5.3%)	28/338 (8.3%)	0/66 (0.0%)
Acute kidney injury	63/310 (20.3%)	11/94 (11.7%)	11/160 (6.9%)	63/244 (25.8%)	74/338 (21.9%)	0/66 (0.0%)
Thrombocytopenia	28/310 (9.0%)	4/94 (4.3%)	10/160 (6.3%)	22/244 (9.0%)	26/338 (7.7%)	6/66 (9.1%)
Intubation	14/310 (4.5%)	8/94 (8.5%)	8/160 (5.0%)	14/244 (5.7%)	22/338 (6.5%)	0/66 (0.0%)

The linezolid group recorded zero events for ICU admission, septic shock, acute kidney injury, intubation, and 28-day mortality. These zero counts reflect preferential use of linezolid in lower-acuity patients (confounding by indication) and should not be read as a protective effect

MIC distributions for key *anti-enterococcal* agents are summarized in Supplementary Table S2. Linezolid MICs were similar between VSE and VRE isolates. Teicoplanin and vancomycin MICs were available only in the VSE group, while daptomycin and tigecycline MICs were primarily available in the VRE group. Among VRE isolates, teicoplanin susceptibility by disk diffusion was used as a phenotypic surrogate for VanB-type resistance.

### Competing-risk analysis of in-hospital mortality (CIF/Gray test)

**Fig. 2:**
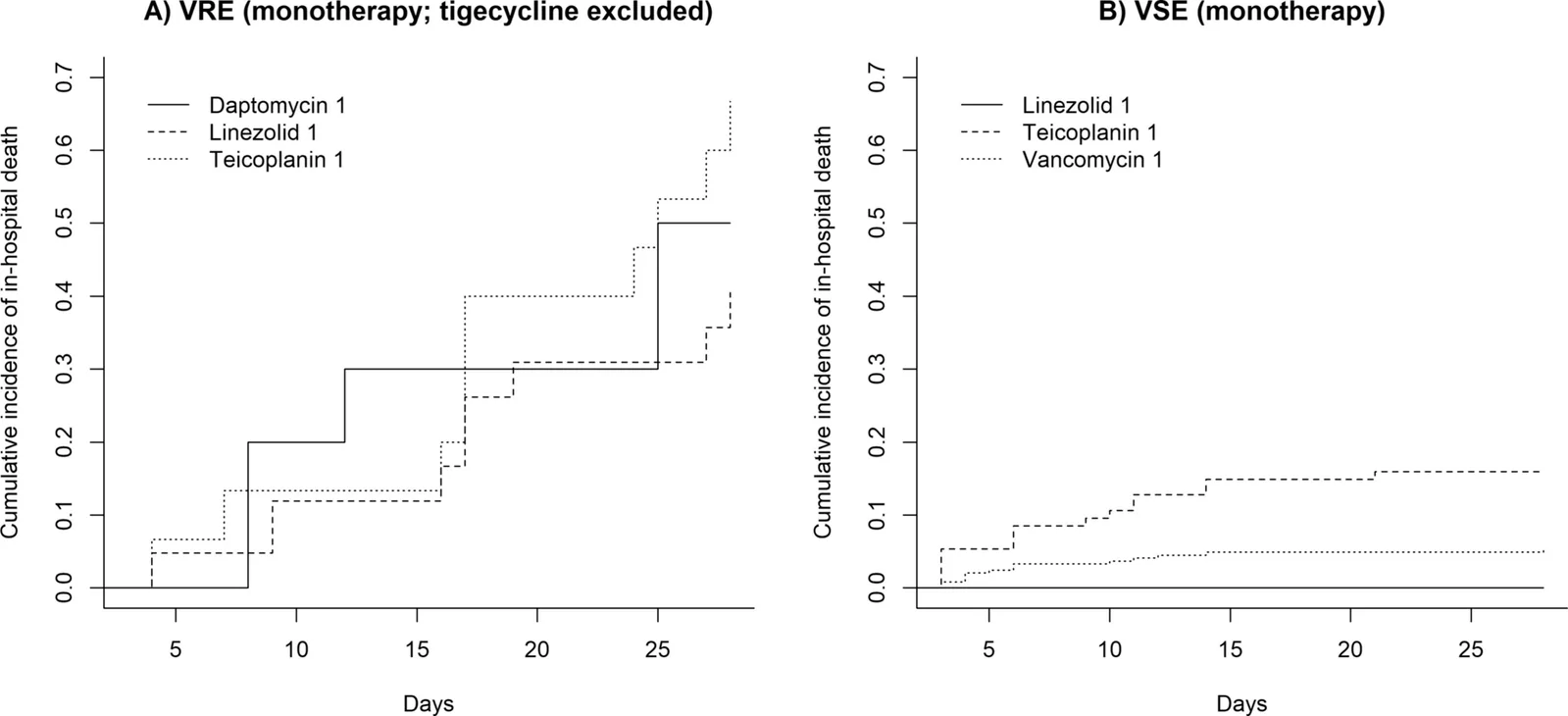
Cumulative incidence of in-hospital death by definitive monotherapy regimen. Cumulative incidence functions (CIFs) for in-hospital death were estimated using a competing-risks approach with discharge alive treated as a competing event. Panels show regimen-specific CIFs within VRE (**A**) (linezolid, teicoplanin, daptomycin; tigecycline excluded) and VSE (**B**) (linezolid, teicoplanin, vancomycin) cohorts, restricted to patients receiving exactly one definitive agent. Gray’s test compared CIFs across regimens (VRE, *p* = 0.246; VSE, *p* < 0.001)

Among VRE bacteremia (Fig. 2A) patients receiving definitive monotherapy excluding tigecycline (*n* = 67), the cumulative incidence of in-hospital death did not differ significantly across linezolid, teicoplanin, and daptomycin (Gray’s test *p* = 0.246), with discharge alive treated as a competing event.

In the VSE cohort (Fig. 2B) receiving definitive monotherapy (*n* = 404), the cumulative incidence of in-hospital death differed significantly across linezolid, teicoplanin, and vancomycin (Gray’s test *p* < 0.001), accounting for discharge as a competing event.

In the adjusted Fine–Gray competing risks model (Table 5), higher qPitt score was independently associated with greater subdistribution hazard of death (sHR 2.21, 95% CI 1.62–3.01; *p* < 0.001). Tigecycline (sHR 4.00, 95% CI 2.11–7.60; *p* < 0.001) and daptomycin exposure (sHR 3.11, 95% CI 1.37–7.07; *p* = 0.007) were associated with higher subdistribution hazard of death, whereas vancomycin exposure was associated with lower subdistribution hazard (sHR 0.36, 95% CI 0.20–0.67; *p* = 0.001). Age, CCI, septic shock, and teicoplanin exposure were not significant.

**Table 5:** Fine–Gray competing risks regression for in-hospital mortality (death as event of interest; discharge alive as competing event) in the complete *Enterococci* cohort (*N* = 501)

Variable	Adjusted sHR	95% confidence interval	*p*-value
Age	1.010	0.992–1.029	0.286
Charlson Comorbidity Index	1.053	0.954–1.163	0.307
Quick Pitts (qPitt) score	2.211	1.623–3.011	< 0.001
Septic shock	1.573	0.764–3.239	0.219
Teicoplanin	1.167	0.665–2.050	0.590
Vancomycin	0.360	0.195–0.667	0.001
Daptomycin	3.111	1.369–7.069	0.007
Tigecycline	4.001	2.105–7.604	< 0.001

*sHR* subdistribution hazard ratio

## Discussion

This study evaluated clinical outcomes of *enterococcal* bloodstream infections, comparing VRE versus VSE bacteremia and describing outcome patterns by antibiotic exposure. A fundamental limitation of this study is confounding by indication: antibiotic selection was driven by illness severity, renal function, drug availability, and cost rather than random assignment, making regimen–outcome associations hypothesis-generating rather than causal (Tholany 2023; Zimmermann 2025). This is particularly evident in our VSE cohort, where linezolid-exposed patients had near-zero adverse clinical endpoints (zero ICU admissions, zero mortality, zero AKI), suggesting strong treatment selection bias rather than a true protective drug effect—linezolid was preferentially reserved for lower-acuity patients without renal impairment or severe sepsis. Conversely, in VRE bacteremia, linezolid is an expected active agent and was used more broadly across severity strata. Similarly, tigecycline-exposed patients demonstrated significantly higher mortality in unadjusted analyses (sHR 4.00, 95% CI 2.11–7.60), likely reflecting its use as salvage therapy in the sickest patients with contraindications to linezolid and no daptomycin access, rather than drug harm per se (Mermel et al. 2009). These treatment-selection patterns underscore that observed regimen–outcome associations should be interpreted with caution and do not establish causality.

In our tertiary-care cohort from Karachi, VRE accounted for 20.4% of *enterococcal* bloodstream infections, differing from earlier US surveillance (2%) and later estimates approaching one-third, while Canadian estimates have been closer to 10% (Hwang et al. 2025; Baddour et al. 2015). These comparisons underscore substantial geographic and temporal variability in VRE prevalence. The urinary tract was the most common presumed source in our cohort, contrasting with other reports identifying primary bacteremia or intra-abdominal sources as most frequent (Echeverria-Esnal et al. 2019). The predominance of urinary tract sources in our cohort may also reflect our exclusion of polymicrobial bacteremia, which can alter the apparent source distribution by removing cases with concurrent intra-abdominal or respiratory co-pathogens.

In our VRE cohort, when accounting for discharge as a competing event using Fine–Gray competing-risks analysis, cumulative incidence of in-hospital death did not differ significantly across linezolid, teicoplanin, and daptomycin monotherapy groups (Gray’s *p* = 0.246). This finding contrasts with unadjusted analyses suggesting linezolid benefit and likely reflects the small sample size (*n* = 67 after excluding tigecycline and combination therapy) and residual confounding despite competing-risks adjustment. Our findings appear to contradict the largest retrospective cohort (Britt et al. 2015), which reported higher mortality with linezolid versus daptomycin in 1201 US veterans with VRE bacteremia (Britt et al. 2015). However, that study and subsequent meta-analyses predominantly included patients receiving low-dose daptomycin (6–8 mg/kg/day), whereas our institutional protocol employed daptomycin 10 mg/kg/day (Britt et al. 2015; Whang et al. 2013; Balli et al. 2014), consistent with current CLSI recommendations for *E. faecium* bacteremia. Recent prospective data demonstrate that daptomycin efficacy is dose-dependent: in a multicenter cohort of 661 VRE bacteremia patients, each 1 mg/kg increase in daptomycin dose independently predicted lower mortality (aOR 0.85, 95% CI 0.73–0.99), and doses ≥ 11 mg/kg were associated with significantly improved survival compared with 8–10 mg/kg, particularly for isolates with daptomycin MIC ≥ 2 mg/L. Similarly, Chuang et al. reported that daptomycin ≥ 9 mg/kg was associated with significantly lower 14-day mortality compared with < 7 mg/kg (aOR 10.57, 95% CI 2.25–49.62). A recent study comparing high-dose daptomycin (≥ 8 mg/kg) with linezolid found that daptomycin efficacy was comparable to linezolid only when pharmacodynamic targets (fAUC/MIC > 75) were achieved; failure to achieve this target was associated with significantly higher mortality than linezolid (aOR 2.51, *p* < 0.001), whereas achieving the target showed no difference (aOR 0.97, *p* = 0.91) (Hwang et al. 2025). The 2015 AHA/IDSA Endocarditis Guidelines recommend daptomycin doses of 10–12 mg/kg/day for multidrug-resistant *enterococcal* infections, and our data support this recommendation (Britt et al. 2015).

In the VSE cohort, cumulative incidence of in-hospital death differed significantly across treatment groups (Gray’s *p* < 0.001), driven primarily by the linezolid-exposed group having zero mortality events and the teicoplanin group having numerically higher mortality (16.0% vs 4.2% for non-teicoplanin, *p* < 0.001). However, these findings must be interpreted with extreme caution due to severe confounding by indication. The linezolid-exposed VSE patients had zero events for ICU admission, septic shock, AKI, intubation, and mortality, indicating that linezolid was selectively reserved for the lowest-acuity patients—likely those with contraindications to vancomycin (e.g., severe penicillin allergy, prior vancomycin reactions) but without renal impairment, hemodynamic instability, or severe sepsis. This treatment selection pattern creates structural zeros in outcome counts and precludes meaningful causal inference about linezolid efficacy in VSE bacteremia. Accordingly, the significant Gray’s test result in the VSE cohort is driven by this artifact rather than by drug effect, and we make no claim that linezolid is superior to vancomycin or teicoplanin for VSE bacteremia. Conversely, vancomycin was preferentially avoided in patients with baseline renal impairment to prevent nephrotoxicity, explaining the strong association between vancomycin exposure and AKI (25.8% vs 6.9%, *p* < 0.001) despite vancomycin being the guideline-recommended first-line agent for ampicillin-resistant VSE (Mermel et al. 2009). In the adjusted Fine–Gray model, vancomycin exposure was independently associated with lower subdistribution hazard of death (sHR 0.36, 95% CI 0.20–0.67, *p* = 0.001), likely reflecting its use in patients healthy enough to tolerate potential nephrotoxicity. These findings support prior work suggesting that timely appropriate therapy may be more important than the specific agent selected for VSE bacteremia and align with data showing no significant mortality differences between linezolid and glycopeptides in VSE bacteremia when both are administered appropriately (Bussini et al. 2025; Van Heuverswyn et al. 2023; Russo et al. 2022; Jin et al. 2025). From a stewardship perspective, we discourage routine linezolid use for ampicillin- and vancomycin-susceptible *Enterococcus* when standard first-line options (ampicillin, vancomycin) are appropriate, to reduce unnecessary exposure and selection pressure for linezolid resistance.

Prior studies consistently show that mortality in *enterococcal* bacteremia is driven primarily by comorbidity burden and severity of illness rather than resistance status alone (Zimmermann 2025; Cheah et al. 2013; Del Monte et al. 2025; Serapide et al. 2025; Weber et al. 2019). In our adjusted Fine–Gray competing-risks model, higher qPitt score was the strongest independent predictor of mortality (sHR 2.21, 95% CI 1.62–3.01, *p* < 0.001), underscoring the primacy of baseline severity in determining outcomes. This finding aligns with recent multicenter data showing that Charlson Comorbidity Index > 3 points (*p* < 0.001), Pitt Bacteremia Score ≥ 4 (*p* = 0.031), and septic shock (*p* < 0.001) were independently associated with 30-day mortality in VRE bacteremia, whereas time to targeted therapy < 24 h and surgery for source control were independently associated with survival (Jin 2025). Similarly, a large Swiss cohort found that infectious diseases consultation within 48 h (aHR 0.40, 95% CI 0.28–0.57), appropriate antimicrobial therapy within 48 h (aHR 0.54, 95% CI 0.34–0.86), and source control interventions performed within 48 h (aHR 0.22, 95% CI 0.14–0.36) were independently associated with survival in *enterococcal* bacteremia (Zimmermann 2025). A propensity score-matched cohort of 12,666 US veterans with *enterococcal* bacteremia found that ID consultation was associated with significantly lower 30-day mortality (OR 0.56, 95% CI 0.50–0.64) and higher rates of appropriate antibiotic use, blood culture clearance documentation, and echocardiography (Tholany 2023). We did not systematically capture ID consultation, time to appropriate therapy, or source control interventions in our cohort, representing a major limitation and potential source of residual confounding in our treatment comparisons.

Catheter-associated infection was significantly more common in VRE than VSE bacteremia (38.1% vs 16.1%, *p* < 0.001), aligning with reports showing that central line presence and delayed removal correlate with increased mortality (Zimmermann 2025; Mermel 2009). The 2009 IDSA CLABSI Guidelines recommend removal of short-term catheters for *enterococcal* CLABSI and removal of long-term catheters in cases of sepsis, persistent bacteremia > 72 h, or endocarditis Mermel 2009. Recent data from cancer patients with *enterococcal* CLABSI suggest that early catheter removal (< 3 days) may be associated with better outcomes when removal is clinically indicated, though catheter retention beyond 7 days was also associated with high success rates in selected patients (Awadh et al. 2021). A recent Chinese ICU cohort found that early central venous catheter removal (HR 0.01, 95% CI < 0.001, *p* < 0.001) and early source control (HR 0.08, 95% CI < 0.001, *p* < 0.001) were among the strongest independent predictors of survival in *enterococcal* bacteremia, whereas delayed antimicrobial therapy > 24 h (HR 11.11, *p* < 0.001) significantly increased mortality risk (Jin 2025). For uncomplicated *enterococcal* CLABSI with catheter removal, a 7-day treatment course appears safe and is not associated with higher 30-day mortality compared with longer durations. We did not capture catheter removal rates or timing of removal in our cohort, which may have confounded our treatment-outcome associations, as patients with early source control may have had better outcomes regardless of antibiotic choice. Future studies should systematically capture ID consultation, time to appropriate therapy, and source control interventions, including catheter removal timing—as these factors may be more important determinants of survival than antibiotic choice alone (Zimmermann 2025; Jin 2025; Serapide et al. 2025).

Although we defined exposure based on antibiotics initiated within 48–72 h of index blood culture collection to minimize immortal time bias, we did not systematically analyze time to appropriate therapy as a predictor of mortality. Recent evidence suggests that the impact of early appropriate therapy may differ between VRE and VSE bacteremia. In a large multicenter Italian study of VRE bacteremia, time to targeted therapy < 24 h was independently associated with 30-day survival (*p* = 0.006), and administration of early effective therapy within 48 h was associated with mortality rates < 33% (Russo 2022; Serapide et al. 2025). Similarly, a recent Taiwanese study found that among VRE bacteremia patients requiring empiric Gram-positive coverage, empiric anti-VRE therapy (linezolid or daptomycin) was independently associated with lower 28-day mortality compared with empiric glycopeptides (aOR 0.41, 95% CI 0.17–0.98, *p* = 0.046), even though both groups eventually received VRE-active antibiotics; each day’s delay in VRE-active therapy correlated with higher mortality (0 days, 37.0%; 1 day, 57.7%; ≥ 2 days, 64.5%; *p* = 0.02) (Ou et al. 2025). However, in a large multicenter study of unselected *enterococcal* bacteremia (predominantly VSE), early *anti-enterococcal* empirical therapy within 48 h did not reduce 14- or 30-day mortality in adjusted analyses, except in the subgroup with VRE bacteremia (Bussini et al. 2025). These findings suggest that early targeted therapy may be particularly critical in VRE bacteremia, where delays in active therapy directly translate to prolonged exposure to ineffective glycopeptides, whereas in VSE bacteremia, most empiric regimens (vancomycin, piperacillin-tazobactam) provide adequate coverage. The absence of time-to-therapy data in our study limits our ability to disentangle the effects of antibiotic choice from timeliness of appropriate therapy.

This study has several important limitations. First, we were unable to reliably distinguish *E. faecalis* from *E. faecium* in most cases, which is a major limitation because these species differ substantially in baseline mortality risk (*E. faecium* has approximately twofold higher mortality than *E. faecalis* in VRE bacteremia), resistance profiles, and treatment responses (Del Monte et al. 2025; Vergis et al. 2001). Its retrospective, single hospital-system design limits generalizability. Treatment assignment was not randomized; antibiotic selection was heavily influenced by illness severity, renal function, drug availability, and cost, making regimen–outcome associations susceptible to confounding by indication and hypothesis-generating rather than causal. Our models omitted several established predictors of mortality, including infectious diseases consultation, time to appropriate therapy, source control interventions (Table 6), catheter removal timing, and Charlson Comorbidity Index, which may have resulted in residual confounding. Several exposure groups were small (particularly teicoplanin and daptomycin), resulting in wide confidence intervals and limited power. Despite these limitations, this cohort provides pragmatic, real-world data on outcomes across commonly used regimens for both VRE and VSE bacteremia in a resource-limited setting.

**Table 6:** Key antimicrobial stewardship priorities for *enterococcal* bacteremia in resource-limited settings

Priority	Recommendation	Evidence level	References
1. Early ID consultation	Obtain infectious diseases consultation within 48 h of positive blood culture	Strong	(Furuichi 2018; Kattan 2020)
2. Prompt appropriate therapy	Initiate appropriate antibiotic therapy within 48 h of blood culture positivity	Strong	(Russo 2022; Serapide et al. 2025)
3. High-dose daptomycin for VRE	Use daptomycin ≥ 9 mg/kg/day (preferably ≥ 11 mg/kg/day) when treating VRE bacteremia	Strong	(Ohnuma 2023; Mejia-Chew et al. 2019)
4. Early source control	Remove central venous catheters 3 days when clinically indicated for catheter-associated infection	Moderate	(Field et al. 2014; Höfler 2018)
5. Avoid tigecycline for VRE BSI	Do not use tigecycline for VRE bloodstream infection when linezolid or daptomycin are available	Strong	(Mermel et al. 2009; Cattaneo et al. 2021)
6. Avoid linezolid for susceptible isolates	Reserve linezolid for ampicillin-resistant, vancomycin-resistant isolates; use ampicillin or vancomycin for susceptible strains	Moderate	(Rosselli Del Turco 2021)

## Conclusion

This cohort showed that in a resource-limited country, VRE bacteremia was associated with more severe symptoms at onset and was more often catheter-related than VSE. Differences in VRE definitive monotherapy among regimens, in terms of discharge as a competing event, did not reach statistical significance. In contrast, differences in VSE were strongly confounded by indication and by the structural limitation of no events. The only factor found across all cases to independently predict death was illness severity (qPitt score), clearly demonstrating that the first and most important determinants of outcome are the initial clinical condition and the quality of patient care. Exposure to tigecycline was also independently associated with increased risk of death, which likely reflects its use primarily as a “salvage” agent in those who have been sick for an extended period; therefore, it is prudent to avoid using tigecycline as a therapeutic option for patients with VRE bacteremia if there are other viable options available. These results also provide additional evidence supporting a stewardship approach that emphasizes prompt consultation with specialists, timely administration of an effective antibiotic regimen, and source control as key elements of appropriate antibiotic stewardship. Lastly, these results highlight the need for future prospective studies with species-specific pathogen identification and the standardization of treatment and care processes.

## Supplementary information

Below is the link to the electronic supplementary material.

ESM 1 (DOCX 14.4 KB)

ESM 2 (DOCX 16.3 KB)

## Data Availability

The datasets used and/or analyzed during the current study are available from the corresponding author on reasonable request.
