# Supplementary material for: Outcomes of linezolid, glycopeptides, and other regimens in ampicillin-resistant, vancomycin-susceptible and vancomycin-resistant enterococcal bacteremia in a resource-limited setting: stewardship implications
Source: Naunyn Schmiedebergs Arch Pharmacol. 2026 Jul 12;399(13):20945–57. doi: 10.1007/s00210-026-05701-2 (PMC13695658; doi:10.1007/s00210-026-05701-2)
Supplement: Supplementary file 1 [file 210_2026_5701_MOESM1_ESM.docx]

**Table S1. VRE cohort (n=97): Outcomes by tigecycline exposure (salvage)**

| **Outcome** | **No tigecycline (n=67)** | **Tigecycline (n=30)** | **P value** |
| --- | --- | --- | --- |
| **28-day mortality** | 32 (47.8%) | 24 (80.0%) | 0.003 |
| **AKI** | 10 (14.9%) | 8 (26.7%) | 0.169 |
| **Septic shock** | 10 (14.9%) | 8 (26.7%) | 0.169 |
| **ICU admission** | 48 (71.6%) | 25 (83.3%) | 0.217 |
| **Intubation** | 29 (43.3%) | 18 (60.0%) | 0.128 |
| **Thrombocytopenia** | 11 (16.4%) | 0 (0.0%) | 0.016 |
