# Supplementary material for: Outcomes of linezolid, glycopeptides, and other regimens in ampicillin-resistant, vancomycin-susceptible and vancomycin-resistant enterococcal bacteremia in a resource-limited setting: stewardship implications
Source: Naunyn Schmiedebergs Arch Pharmacol. 2026 Jul 12;399(13):20945–57. doi: 10.1007/s00210-026-05701-2 (PMC13695658; doi:10.1007/s00210-026-05701-2)
Supplement: Supplementary file 2 [file 210_2026_5701_MOESM2_ESM.docx]

| **Antibiotic** | **Cohort** | **Valid N** | **Missing N** | **Mean ± SD** | **MIC50** | **MIC90** | **Range** | **MIC distribution (n, %) among valid** |
| --- | --- | --- | --- | --- | --- | --- | --- | --- |
| Linezolid | VSE (n=404) | 300 | 104 | 0.472 ± 0.214 | 0.50 | 1.00 | 0–1.00 | 0: 1 (0.3%); 0.25: 94 (31.3%); 0.50: 174 (58.0%); 1.00: 31 (10.3%) |
| Linezolid | VRE (n=97) | 97 | 0 | 0.487 ± 0.189 | 0.50 | 0.50 | 0–1.00 | 0: 1 (1.0%); 0.25: 19 (19.6%); 0.50: 69 (71.1%); 1.00: 8 (8.2%) |
| Teicoplanin | VSE (n=404) | 290 | 114 | 0.335 ± 0.119 | 0.25 | 0.50 | 0.25–0.50 | 0.25: 191 (65.9%); 0.50: 99 (34.1%) |
| Teicoplanin | VRE (n=97) | 0 | 97 | — | — | — | — | Not available |
| Vancomycin | VSE (n=404) | 290 | 114 | 0.550 ± 0.150 | 0.50 | 0.50 | 0.50–1.00 | 0.50: 261 (90.0%); 1.00: 29 (10.0%) |
| Vancomycin | VRE (n=97) | 0 | 97 | — | — | — | — | Not available |
| Daptomycin | VSE (n=404) | 0 | 404 | — | — | — | — | Not available |
| Daptomycin | VRE (n=97) | 10 | 87 | 1.000 ± 0.000 | 1.00 | 1.00 | 1.00–1.00 | 1.00: 10 (100%) |
| Tigecycline | VSE (n=404) | 0 | 404 | — | — | — | — | Not available |
| Tigecycline | VRE (n=97) | 30 | 67 | 0.144 ± 0.082 | 0.190 | 0.190 | 0.023–0.250 | 0.023: 9 (30.0%); 0.190: 19 (63.3%); 0.250: 2 (6.7%) |

**Table S2. MIC summary by VRE/VSE status**
